# A novel lncRNA-mediated epigenetic regulatory mechanism in periodontitis

**DOI:** 10.7150/ijbs.87977

**Published:** 2023-10-09

**Authors:** Zoe Xiaofang Zhu, Yao Liu, Jinghao Wang, Ying Xie, Rachel Yuantong Li, Qian Ma, Qisheng Tu, Neiman A Melhem, Sandrine Couldwell, Rady E. El-Araby, Albert Tai, Thomas E. Van Dyke, Nadeem Karimbux, Y. Natalie Jeong, Jake Jinkun Chen

**Affiliations:** 1 Division of Oral Biology, Tufts University School of Dental Medicine, 136 Harrison Ave, M&V Building Room 830, Boston, MA 02111, United States.; 2 Department of Periodontology, Tufts University School of Dental Medicine, Boston, MA, 02211, United States.; 3 State Key Laboratory of Oral Diseases & National Clinical Research Center for Oral Diseases & Department of Oral and Maxillofacial Surgery, West China Hospital of Stomatology, Sichuan University, Chengdu, 610041, Sichuan, China.; 4 Department of Immunology, Tufts University School of Medicine, Boston, MA, United States.; 5 Data Intensive Studies Center, Tufts University, Medford, MA, United States.; 6 Clinical and Translational Research, Oral Medicine, Infection, and Immunity, Harvard School of Dental Medicine, Forsyth Institute, Boston, MA, United States.; 7 Department of Genetics, Molecular and Cell Biology, Tufts University School of Medicine, Tufts School of Graduate Biomedical Sciences, 136 Harrison Ave, M&V Room 811, Boston, MA 02111, United States.

**Keywords:** periodontitis, long-noncoding RNA, single-cell RNA sequencing, immune response, bone loss

## Abstract

Periodontitis is a highly prevalent chronic inflammatory disease with an exaggerated host immune response, resulting in periodontal tissue destruction and potential tooth loss. The long non-coding RNA, LncR-ANRIL, located on human chromosome 9p21, is recognized as a genetic risk factor for various conditions, including atherosclerosis, periodontitis, diabetes, and cancer. LncR-APDC is an ortholog of ANRIL located on mouse genome chr4. This study aims to comprehend the regulatory role of lncR-APDC in periodontitis progression. Our experimental findings, obtained from lncR-APDC gene knockout (KO) mice with induced experimental periodontitis (EP), revealed exacerbated bone loss and disrupted pro-inflammatory cytokine regulation. Downregulation of osteogenic differentiation occurred in bone marrow stem cells harvested from lncR-APDC-KO mice. Furthermore, single-cell RNA sequencing of periodontitis gingival tissue revealed alterations in the proportion and function of immune cells, including T and B cells, macrophages, and neutrophils, due to lncR-APDC silencing. Our findings also unveiled a previously unidentified epithelial cell subset that is distinctively presenting in the lncR-APDC-KO group. This epithelial subset, characterized by the positive expression of *Krt8* and *Krt18*, engages in interactions with immune cells through a variety of ligand-receptor pairs. The expression of *Tff2*, now recognized for its role in chronic inflammatory conditions, exhibited a notable increase across various tissue and cell types in lncR-APDC deficient mice. Additionally, our investigation revealed the potential for a direct binding interaction between lncR-APDC and *Tff2*. Intra-gingival administration of AAV9-lncR-APDC was shown to have therapeutic effects in the EP model. In conclusion, our results suggest that lncR-APDC plays a critical role in the progression of periodontal disease and holds therapeutic potential for periodontitis. Furthermore, the presence of the distinctive epithelial subpopulation and significantly elevated *Tff2* levels in the lncR-APDC-silenced EP model offer new perspectives on the epigenetic regulation of periodontitis pathogenesis.

## Introduction

A recent report from the Centers for Disease Control and Prevention (CDC) shows that 47.2% of American adults over age 30 have periodontal disease (PD) and the incidence of PD rises to 70.1% in adults 65 years and older 1. Based on the high prevalence, PD is considered a common public health problem. The basic pathology of PD is the immuno-inflammatory host response against commensal bacteria and excessive alveolar bone resorption leading to tooth loss 2. Furthermore, PD can trigger general systemic inflammation, adversely influencing cardiovascular, neural, reproductive, and endocrine systems 3-5. As a complex chronic inflammatory and immune disease, the molecular mechanisms of PD are not fully understood yet. The primary goal of periodontitis treatment is to prevent the progression of the disease, manage its symptoms, and restore the health of the gingiva and supporting structure of the teeth. Periodontitis treatment typically involves both non-surgical and surgical therapies 6. A pressing need exists for an innovative treatment that blocks the major elements of host mediated pathogenesis and stimulates endogenous regeneration.

Noncoding RNAs (ncRNAs) are a large segment (more than 80%) of the transcriptome that lack apparent protein encoding capability but are functionally important. Long noncoding RNAs (lncRNAs) are a subgroup of ncRNAs with a length of more than 200 nucleotides. Emerging evidence suggests that lncRNAs participate in a wide repertoire of genome organization and life processes such as growth and development, cell proliferation, differentiation, immune response, and certain diseases, such as cancer and cardiovascular diseases 7,8.

LncRNA ANRIL (short as ANRIL), situated on chromosome 9p21.3 in humans, is a lncRNA encoded in the opposite direction within the INK4/ARF locus. ANRIL is the antisense lncRNA of cyclin-dependent kinase inhibitor 2A (CDKN2A) and CDKN2B, both of which are protein coding genes situated in the INK4 locus. ANRIL has been proven to be a shared risk factor for atherosclerosis, periodontitis, diabetes, and cancer 9-11. ANRIL is the best replicated genetic locus of atherosclerosis-associated coronary artery disease (CAD) and PD 12,13. The core risk haplotype shared between CAD and PD is located at the 3' end of ANRIL, which implies ANRIL is a prime functional candidate involved in the risk mediating mechanism 14. It can modulate genes and pathways related to inflammation, cell cycle regulation, and atherosclerosis. Additionally, studies suggest a bi-directional association between periodontitis, diabetes, and CAD 5,9,15. For example, Individuals with diabetes have an increased likelihood of developing periodontitis and those with both periodontitis and diabetes tend to exhibit poorer glycemic control. Thus, there are strong correlations among the three chronic inflammatory diseases: atherosclerosis, PD, and diabetes. ANRIL, as a shared risk factor in these conditions, likely plays a pivotal role in their crosstalk, specifically in how these diseases mutually influence each other through the regulatory effect of ANRIL. The PD-related ANRIL studies have reported that ANRIL is remarkably lower in the peripheral blood of PD patients in Iran 16 and significantly under-expressed in the inflamed gingival tissue in European populations 17. Downregulated ANRIL inhibits the osteogenic differentiation of periodontal ligament cells (PDLCs) 18. In addition, it was also shown that elevated high sensitive C-reactive protein (hsCRP), a marker for systemic inflammation and a risk marker for periodontitis, was significantly associated with ANRIL SNP rs1333048 19. These studies suggest the potential of lncRNAs as diagnostic biomarkers and targets for the treatment of PD.

We know the disruption of the homeostatic balance of bone remodeling and inflammation both play crucial roles in PD development. However, how ANRIL regulates bone regeneration and inflammation remains unclear. Therefore, our study intentionally focused on the AK148321 region (i.e., mouse Gm12610), an orthologous counterpart to human ANRIL on mouse chromosome 4, to delve into ANRIL's regulatory role and therapeutic potential in the context of PD using a mouse model. We deliberately selected this region based on its conserved genomic homology with the human ANRIL locus, ensuring meaningful translatability to the human condition. Additionally, this region's relevance to PD and the capacity to leverage a genetically modified animal model (i.e., the chr4 Δ70kb/Δ70kb mouse model), allowed for a thorough investigation into ANRIL's functional implications. By utilizing this region, we aimed to assess the therapeutic impact of ANRIL modulation, providing crucial insights into potential therapeutic strategies that target PD. We coined the term lncRNA-APDC (APDC) for the AK148321 region, reflecting its major functions in atherosclerosis, periodontitis, diabetes, and cancer. APDC maintained the synteny of ANRIL with its neighboring genes *Cdkn2a*, *Cdkn2b*, and methyl-thioadenosine phosphorylase (*Mtap*) 20, and preserved its functions as mitigating neuroinflammation and atherosclerosis 21,22. Inflammation and uncoupling of bone formation and resorption were studied in both APDC deficient and wild-type mice with and without periodontitis. The objective of this study was to discover the preventive and/or therapeutic impact of APDC in periodontitis and elucidate the underlying molecular mechanisms of action.

## Results

### APDC deficiency aggravates bone loss and inflammation caused by periodontitis

**Figure 1:**
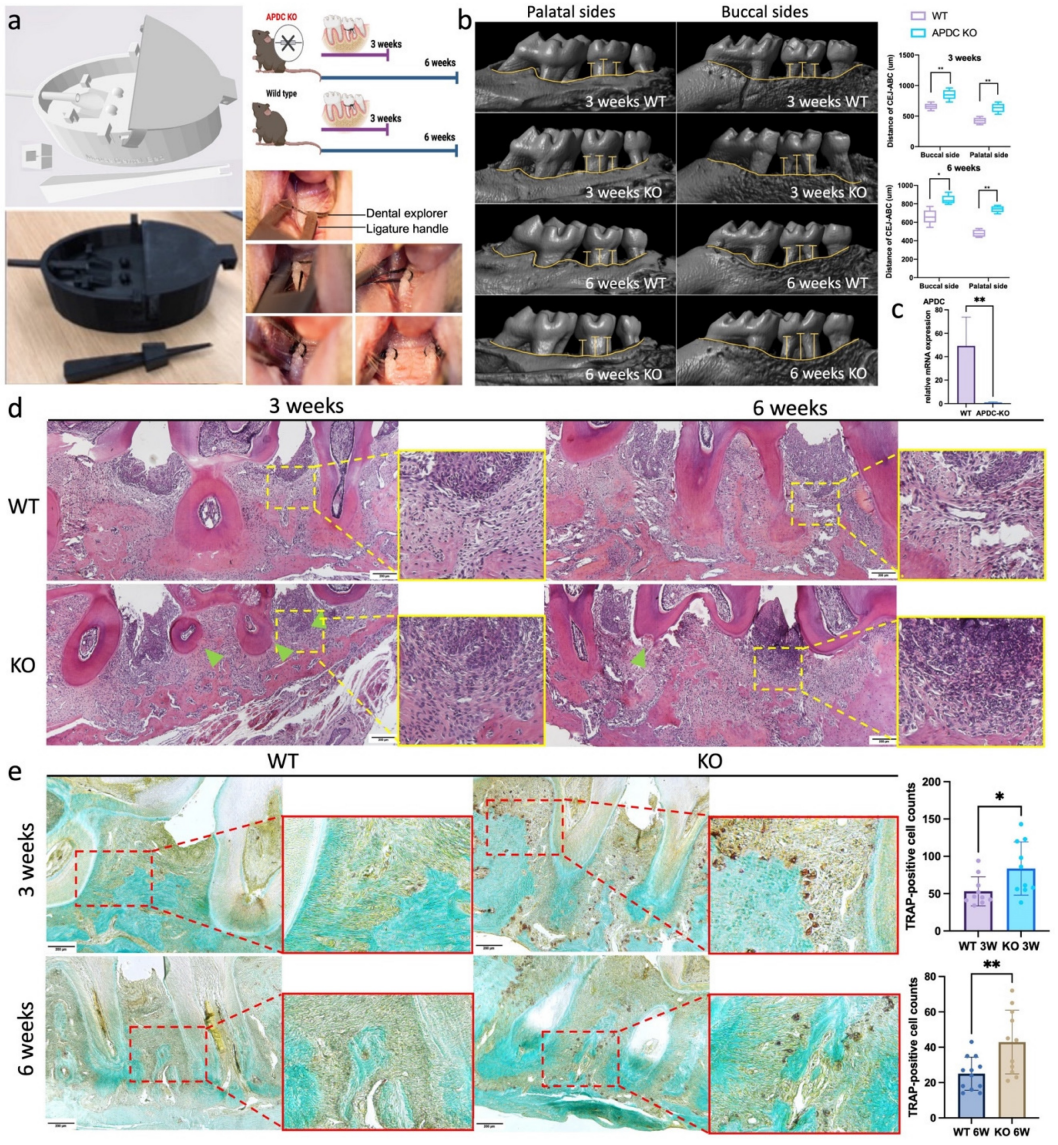
EP model on APDC deficiency mice and control mice. a) The digital (.stl) file and 3D printed copy of the surgical tools. b) Morphology analysis showed the distance from the alveolar bone crest (ABC) to the cementoenamel junction (CEJ) on the buccal sides and palatal sides (n=7). c) the expression level of APDC in wildtype and APDC KO mouse. d) HE staining of the periodontal tissues. The zoom-ins show the inflamed periodontal tissues. Green triangles show cementum resorption (discontinuous cementum). e) TRAP staining shows the TRAP positive cells (red) on the alveolar bone surface in APDC KO and control group at 3- and 6- weeks. Original magnification 400×. Scale bars = 200 μm. Values are shown as mean± SD. * p < 0.05, **p < 0.01.

The experimental periodontitis (EP) model was performed on 4-month-old female APDC-KO and wild-type mice with 3D printed surgical tools (Fig. 1a). Ligatures were placed surrounding the second molar on both sides of the maxilla for 3 weeks and 6 weeks (n = 8-10 per group). Micro-CT reconstruction showed that the distance from the cementoenamel junction to the alveolar bone crest (CEJ-ABC) was significantly increased in the APDC-KO groups 1 by 29% on the buccal side and 49% on the palatal side at 3 weeks, and 2 by 27% on the buccal side and 54% on the palatal side at 6 weeks compared to the control groups at the same time points (Fig. 1b). These changes indicate more severe alveolar bone resorption in the KO group during different stages of periodontitis development. The successful deletion of APDC was confirmed by the expression level of APDC in KO and wildtype mice (Fig. 1c). H&E histology staining demonstrated intense inflammatory cell infiltrate in the tissue subjacent to the periodontal pocket, exacerbated bone resorption of the alveolar crest between the molars and cementum resorption (i.e., discontinuous cementum) in the APDC-KO group at both 3 and 6 weeks (Fig. 1d). The number of tartrate resistant acid phosphatase (TRAP) positive cells on the alveolar bone surface remarkably increased in the APDC-KO groups compared to the control group at the same time points (Fig. 1e). Taken together, the findings reveal the impact of APDC deficiency on alveolar bone loss and inflammatory infiltrate in periodontal tissues.

### APDC plays a regulatory role in bone metabolism

**Figure 2:**
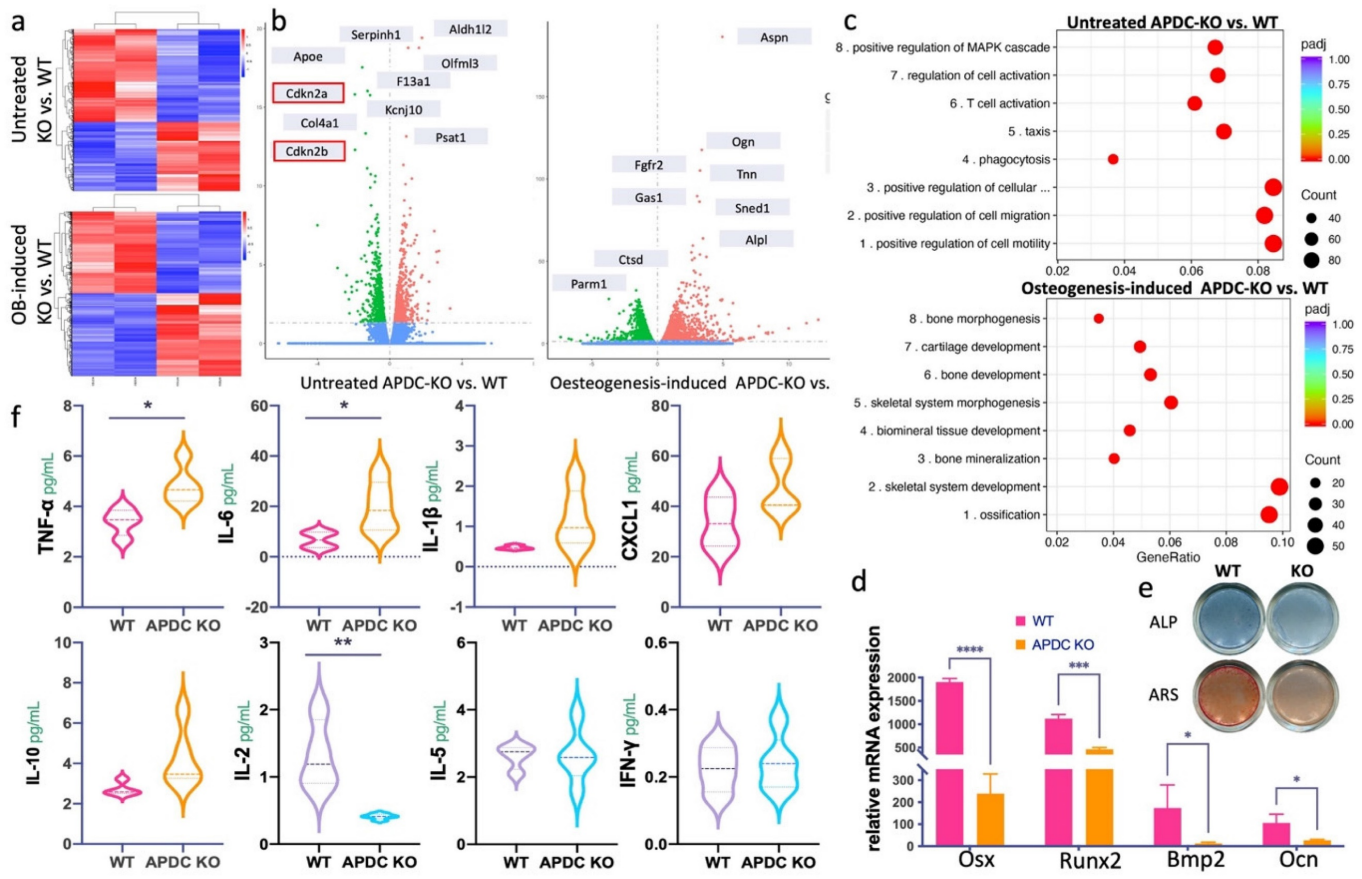
The impact of APDC on inflammation related cytokines and bone metabolism. a) the heatmap of untreated (day0) BMSCs (upper) and osteogenic differentiated (day3) BMSCs (lower) comparing between APDC KO and control mice. b) the volcano plot with top genes marked (WT vs. KO). c) the GO enrichment results at untreated (upper) and osteogenesis-induced (lower) groups. d) the mRNA expression level of the osteogenic markers. e) ALP and ARS staining results (upper) and the mRNA expression level of osteogenesis related genes (lower) for APDC KO and control mice. f) the serum level of cytokines (IL-1β, IL-6, CXCL1, TNF-α, IL-10, IL-2, IL-5 and IFN-γ) (n=5). Values are shown as mean± SD. * p < 0.05, ***p < 0.001, ****p < 0.0001.

To determine the direct impact of APDC on the homeostatic balance of bone remodeling, we performed bulk RNA sequencing on untreated and osteogenic differentiated bone marrow mesenchymal stem cells (BMSCs). The overall gene profiling of untreated and osteogenic differentiated APDC-KO and wildtype BMSCs are presented in a heatmap (Fig. 2a). The top differentially expressed genes (DEG) are marked on the volcano plots (Fig. 2b). Interestingly, *Cdkn2a* and *Cdkn2b* are among the top DEGs in the untreated BMSCs. These results are the first data obtained regarding *Cdkn2a* and *Cdkn2b* expression levels in BMSCs of APDC knockout mice, and they are consistent with the observations by other researchers on other tissue types 20,22. The GO (gene ontology) analysis of untreated KO, compared with wild-type BMSCs, shows that downregulated DEGs primarily pertain to the following biological processes: cell motility and migration, T lymphocytes (T cell) and leukocyte activation, as well as differentiation and activation of the immune response. Conversely, in osteogenic-differentiated groups, APDC deficiency impacts the gene functions of ossification, skeletal system development and morphogenesis, bone mineralization and osteoblast differentiation (Fig. 2c). Furthermore, our *in vitro* BMSCs differentiation showed that the osteogenic markers, including *Osx*, *Runx2*, *Bmp2* and *Ocn*, are remarkably downregulated in the APDC-KO group (Fig. 2d). ALP staining, which marks hypertrophic cells before mineralization onset, and ARS staining, which shows calcium‐rich deposits in cells, were both decreased in the APDC-KO group (Fig. 2e). These results suggest that APDC deficiency disturbs immune response and bone metabolism.

### APDC silencing dysregulates the expression of pro-inflammatory and anti-inflammatory cytokines in the host immune response of periodontitis

A wide range of pro-inflammatory and anti-inflammatory cytokines produced by immune cells play a vital role in initiating and regulating the inflammatory process of periodontitis 23,24. The activation of the acquired immune response by a periodontal pathogen can interfere with bone coupling by stimulating osteoclastogenesis and limiting the formation of new bone following resorption 25, ultimately resulting in alveolar bone loss. We utilized an immunoassay to determine the cytokines involved in the innate and adaptive immune responses of periodontitis. The pro-inflammatory cytokines TNF-α and IL-6 were increased in the APDC-KO mice after 3 weeks of periodontitis induction, whereas the pleiotropic cytokine IL-2, which mediates both pro- and anti-inflammatory functions, was significantly decreased (Fig. 2f).

### Performing single-cell RNA sequencing to identify cell types in the gingival samples

**Figure 3:**
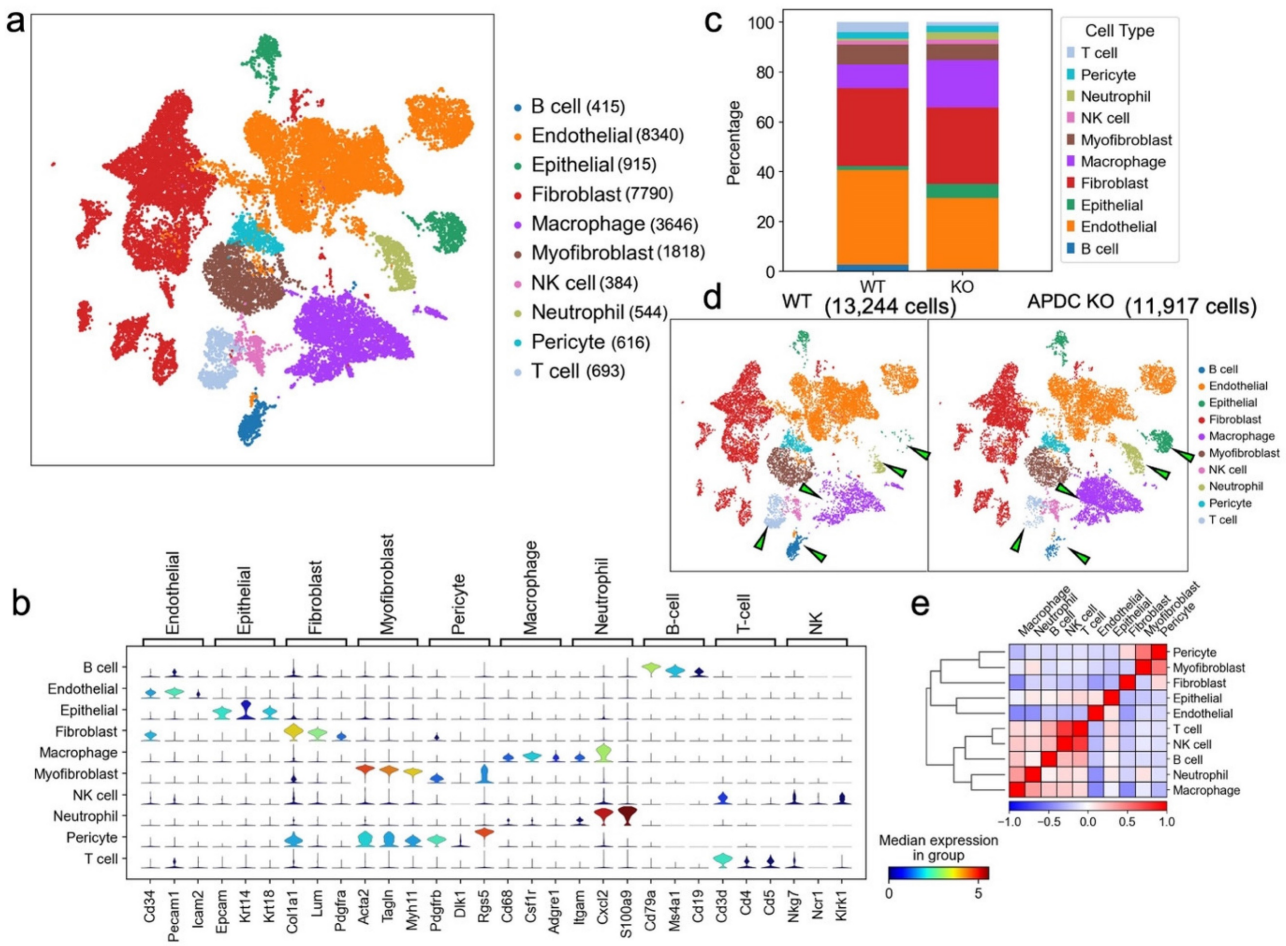
Overview of the 25,161 single cells from periodontal tissue of APDC-KO mice and wildtype mice (n=4 per group). a) Uniform Manifold Approximation and Projection (UMAP) representation of the 25,161 cells, colored by cell type annotation. b) stacked violin plots showing the expression scores of selected marker gene sets across all 10 clusters. c) the bar plots indicating the percentage of each cell type. d) UMAP plot representation the distribution of 11,917 cells of WT group (left) and 13,244 cells of APDC-KO group (right) from the gingival tissue in the 10 clusters. e) the correlation matrix of the 10 cell types.

To uncover the mechanism of APDC's impact on periodontitis at single-cell resolution, we collected gingival tissue surrounding upper-second molars from 4-month-old female APDC-KO and wild-type mice 10 days after ligature placement. The samples underwent dissociation, yielding single cells with a viability >90% which were then processed using barcoding, reverse transcription, cDNA amplification, library construction, and sequencing. The raw sequencing data was aggregated in Cell Ranger. Data analysis and visualization were performed in SCANPY 26 on 25,161 single-cell transcriptomes after data quality filtering. We performed unbiased clustering of single cells using marker genes from the published literature 27,28 and SCSA, a tool based on a score annotation model 29. The resulting cells were annotated into 10 clusters (Fig. 3a). The marker genes for each cluster and their expression scores are presented in stacked violin plots (Fig. 3b). The correlations of annotated cell types are presented in a correlation matrix with a dendrogram (Fig. 3e). This matrix is the mathematical representation that reflects the biological correlations between cell types. The consistency of the correlation pattern and the known features of different cell types (e.g., gene profiles, functions, and shared precursors) confirmed the accuracy of the annotation results. For example, in our samples, the annotated immune cells, including T cells, NK cells, B cells, macrophages and neutrophils, have strong correlations compared to the other cell types. Intriguingly, the inflamed gingival samples of APDC-KO mice showed significant differences in the distribution and proportion of multiple cell types, in contrast to the wild-type control group, even though both groups received identical treatment. Notable alterations included a significant increase in macrophages, neutrophils, and epithelial cells, along with reduced populations of T and B lymphocytes in the APDC deficient mice (Fig. 3c and d).

### The imbalance within the T cell population is due to the lack of CD8+ cytotoxic T cells

**Figure 4:**
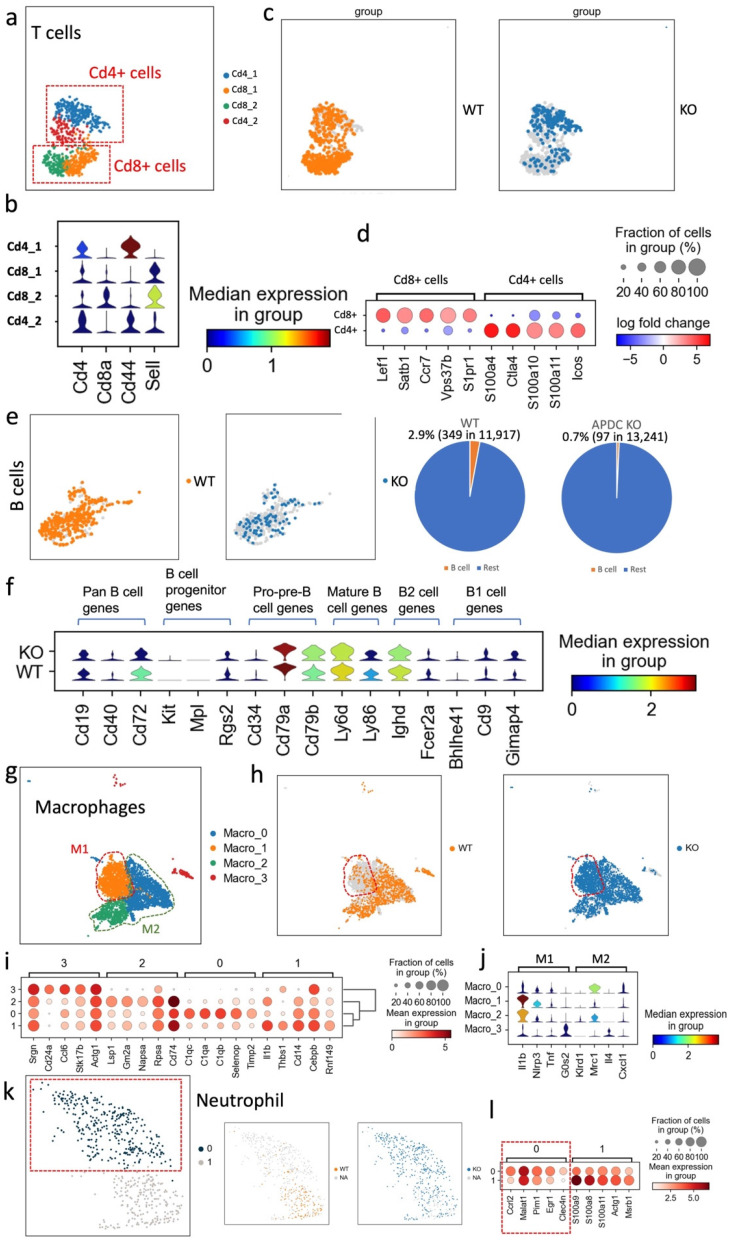
The proportion and function changes of the immune cells in the periodontitis gingival tissue due to the APDC deficiency. (a-d) T cell: a) the subclusters of T cells. b) the violin plots showed the expression of the marker genes. c) the WT and KO group of T cells. d) the DEGs of CD4+ and CD8+ cells. e-f B cell. (e-f) B cell: e) the UMAP (left) and pie chart (right) showed the proportion of WT and KO B cells. f) the violin plots showed the gene expression level of KO and WT at different stages of B cells. (g-j) Macrophage: g) the subclusters of macrophages. h) the WT and KO group of macrophages. i) the highly expressed genes of each subcluster. j) the M1 and M2 macrophages marker genes expression in the subclusters. (k-l) Neutrophil: k) the UMAP of all neutrophils and divided by WT and KO. l) dot plot showed highly expressed genes of cluster 1 and 0.

The predominant immune cell types observed in gingival tissue within 10 days after ligature placement are neutrophils, macrophages, and T cells 30. The T cell is one of the major types of immune cells involved in the host defense and control of immune-mediated periodontitis development. T cells have two main populations, CD4^+^ T cells (i.e., helper or inducer) and CD8^+^ T cells (i.e., cytotoxic or suppressor), and can be further categorized as memory or naïve based on the expression of Sell and CD44 31. The Cd44^low^Sell^+^ population is considered naïve (T_N_), the Cd44^high^Sell^+^ population is classified as central memory T cells (T_CM_) and the Cd44^high^Sell^-^ cells are categorized as effector and/or effector memory T cells (T_E_/T_EM_) 32. In our data, the T cell cluster was further divided into four subclusters (Fig. 4a). The gene expression level of *Cd4, Cd8a, Cd44* and Sell was utilized to identify the subtypes (Fig. 4b). The T cell cluster was also represented based on different groups (i.e., APDC-KO and wild type; Fig. 4c) and DEGs between Cd4+ and Cd8+ clusters (Fig. 4d). Balancing the functionally different T cell subsets is crucial in immune regulation. Notably, the APDC-KO EP group was found to lack Cd8^+^ cells (see cluster CD8_1 and CD8_2 in Fig. 4b), which have the role of cytotoxicity as well as suppression of excessive immune activation and tissue repairing 33. Furthermore, Cd8^+^ T cells are also involved in bone healing. Studies have demonstrated that both mouse Cd8^+^ T cells and *in vitro* expanded human Cd8^+^ T cells can secrete Wnt10b and promote osteoblastogenesis 33. Additionally, Cd8+ T cells have been shown to confer bone protection by suppressing osteoclastogenesis 34. The reduced presence of Cd8+ T cells in the APDC-deficient group aligns with its phenotype of an exacerbated immune response and increased bone resorption.

### The APDC-KO EP model showed reduced B cell counts

B cells constitute a fundamental element of the adaptive immune system, serving vital roles such as antibody and cytokine production, antigen presentation, and the recently discovered B-T reactions 35. Particularly in the context of PD, B cells produce antibodies directed against periodontal pathogens, effectively curbing the progression of periodontal inflammation 36. In our study, we noted a markedly diminished proportion of B cells across all cell types, with the APDC deficiency group registering a mere 0.7% (97 out of 13,241) compared to the wildtype's 2.9% (349 out of 11,917; Fig. 4e). The low B cell level in inflamed gingival tissue could suggest that the immune system might have difficulty mounting an effective response against the infection. 36. Moreover, using the marker genes of different B cell stages, we determined the proportion and functional level of B cells at various developmental phases (Fig. 4f). Specifically, highly expressed Cd79a, Cd79b, Ly6d and Ly86 indicate more pre-B cells and mature B cells in the wildtype group than in the APDC deficient group. In addition, Cd72 is considered to be a pan B cell marker broadly expressed from pre-B cells to mature B cells 37. Taken together, the B cells in inflamed periodontal tissue with APDC deficiency have lower counts and are less functional during the immune response.

### An increased pro-inflammatory M1 subtype of macrophages occurs in APDC-KO EP mice

Macrophages are the main immune cell population in gingival tissue from day 5 in the EP mouse model 30. We performed sub-clustering on the macrophage population to gain higher resolution and a better understanding of the host inflammatory and immune mechanisms during PD progression. Four subtypes of macrophages were identified as Macro_0, Macro_1, Macro_2 and Macro_3 (Fig. 4g).

Intriguingly, the Macro_1 subcluster was a much larger proportion of the whole macrophage cluster in the APDC-KO group compared to the wild-type (Fig. 4h). Therefore, we further investigated the highly expressed genes and DEGs of each subtype (Fig. 4i). The Macro_0 cluster highly expressed the C1qc, C1qa and C1qb genes. C1q polarizes macrophages toward an anti-inflammatory (M2-like) phenotype 38. The Macro_1 cluster highly expressed Ilb1 and the NOD-like receptor family pyrin domain containing 3 (*Nlrp3*), which is involved in M1 macrophage polarization 39. This subtype shows a strong pro-inflammatory phenotype as an M1 macrophage. The top genes of the Macro_2 cluster are *Lsp1* and *Gm2a*, which are highly expressed in M2 macrophages. The expression of *Tgfb1*, an anti-inflammatory gene, was also higher in the Macro_2 cluster. The Macro_3 cluster represents a monocyte-macrophage cluster rather than mature macrophages, characterized by high expression of the *Cebpb*, *Adgre1*, and *Cd24a* genes. Among these subclusters, the APDC-KO group had a similar subcluster size for the Macro_0,2,3 subclusters, while a significantly larger Macro_1 subcluster was observed (M1 macrophages; Fig. 4j). The increase of M1 macrophages resulted in the total macrophage proportion of whole sample being doubled in the APDC-KO group compared to the wild-type (19.1% vs. 9.4%) as shown in Fig. 3b. Taken together, more M1 macrophages that produce pro-inflammatory cytokines were observed in the APDC-KO EP gingival tissue.

### Aggravated neutrophil recruitment was evident in the APDC KO EP model

Neutrophils are produced and stored in bone marrow, released into the circulation, and continuously recruited to the site of chronic inflammation 40. In PD, neutrophils are significantly enriched and play critical roles in periodontal homeostasis, including phagocytosis, degranulation, cytokine production, and neutrophil extracellular trap (NET) formation 41,42. In our study, we observed a subcluster of neutrophils, with top DEGs of *Ccrl2*, *Egr1* and *Clec4n*, that only appeared in the APDC-KO group (Fig. 4k and l). Ccrl2 is highly expressed in primary neutrophils and plays an important role in neutrophil recruitment 43. Therefore, the higher *Ccrl2*+ neutrophil level indicates provoked and continuing neutrophil recruitment in the APDC-KO EP gingival samples, which is also consistent with the increased counts of total neutrophils in the APDC-KO group compared to the wild-type (3.2% vs. 1%).

### Endothelial cells, fibroblasts, myofibroblasts and pericytes were found in the inflamed condition

Endothelial cells were the biggest cellular component in the inflamed gingival samples. A total of five subclusters were identified Fig. S1a). The endo_1 subcluster, characterized by high expression levels of *Selp*, *Ackr1* and *Sele* (Fig. S1b), is well-documented to be closely associated with immune regulation during the PD development (44, clearly suggesting that the tissue samples are in an inflammatory condition. We further checked the related gene expression and cell counts in both the KO and wild-type groups. There was no significant disparity between the two groups Fig. S1c and d).

Fibroblasts are centrally involved in the wound healing response and remodeling of the periodontal tissue (45. During the remodeling phase of wound healing, a specific subtype of fibroblast known as myofibroblasts may emerge 46. Myofibroblasts may also derive from alternative sources, including mesenchymal stem cells, pericytes, and epithelial cells 47. It was recently reported that pericytes possess multilineage differentiation capacity and can be the source of tissue stem cells and/or progenitor cells, which are similar to the periodontal ligament stem cells 48. In our data, the gene profiling and cell counts for these three cell populations were consistent with other periodontitis related studies and did not show a disparity between the APDC KO group and the wild type Fig. S1e, f and g and Fig. S2.

### A unique subset of epithelial cells (Krt8+, Krt18+, Krt5-, Krt14-) was identified in APDC-KO mice

**Figure 5:**
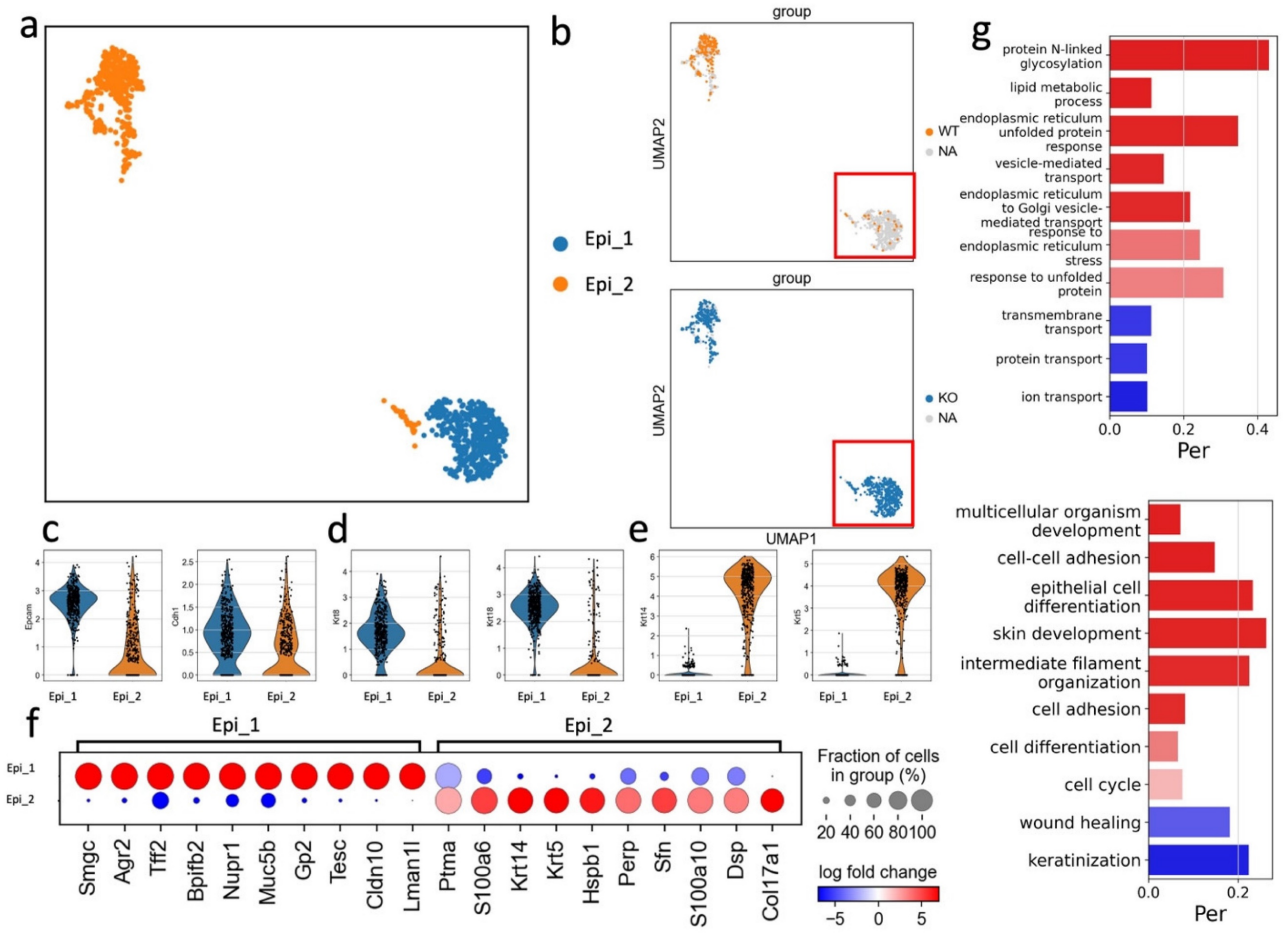
Sub-clustering of the epithelial cells. a) the subclusters of epithelial cells. b) epithelial cells divided by WT and KO (right). c) the classic distinct markers of epithelium. d) Epi_1 cluster highly expressed Krt 8 and Krt 18. e) Epi_2 cluster highly expressed Krt 14 and Krt5. f) the DEGs of Epi_1 and Epi_2. g) the GO enrichment results of Epi_1 and Epi_2.

Gingival epithelial cells act as the first line of defense against periodontal pathogens providing a barrier to bacterial invasion. In this study, we identified two subsets of epithelial cells, both expressing the common epithelial marker genes: *Epcam* and *Cdh1* (Fig. 5a-c). The Epi_2 cluster expressed *Krt14* and *Krt5* and was observed in both APDC-KO and wildtype groups (Fig. 5e). Conversely, the Epi_1 cluster represented a subset of epithelial cells expressing *Krt8* and *Krt18* but devoid of *Krt5* and *Krt14* (Fig. 5d). Krt8 is reported to be involved in the inflammatory response and usually collaborates with Krt18 to regulate protein synthesis and cell movement and inhibit apoptosis 49. Furthermore, the most highly expressed genes in the Epi_1 cluster, including *Agr2*, *Nupr1*, *Muc5b* and *Cldn10* (Fig. 5f), have major functions as barrier maintenance and secretion of mucus and anti-microbial proteins and cytokines. GO enrichment was performed on both clusters, revealing that Epi_2 had typical epithelial-related gene functions, such as cell-cell adhesion and epithelial cell differentiation, whereas Epi_1 had distinct gene enrichment results in protein N-linked glycosylation and lipid metabolic processes. (Fig. 5g). The vast majority of this unique Epi_1 subcluster was from the APDC-KO group (Fig. 5b), suggesting that APDC deficiency alters the gene profiling of epithelial cells in PD, consequently impacting their functions.

### High expression of Trefoil Factor 2 (Tff2) was detected across all cell types in the APDC deficient group

**Figure 6:**
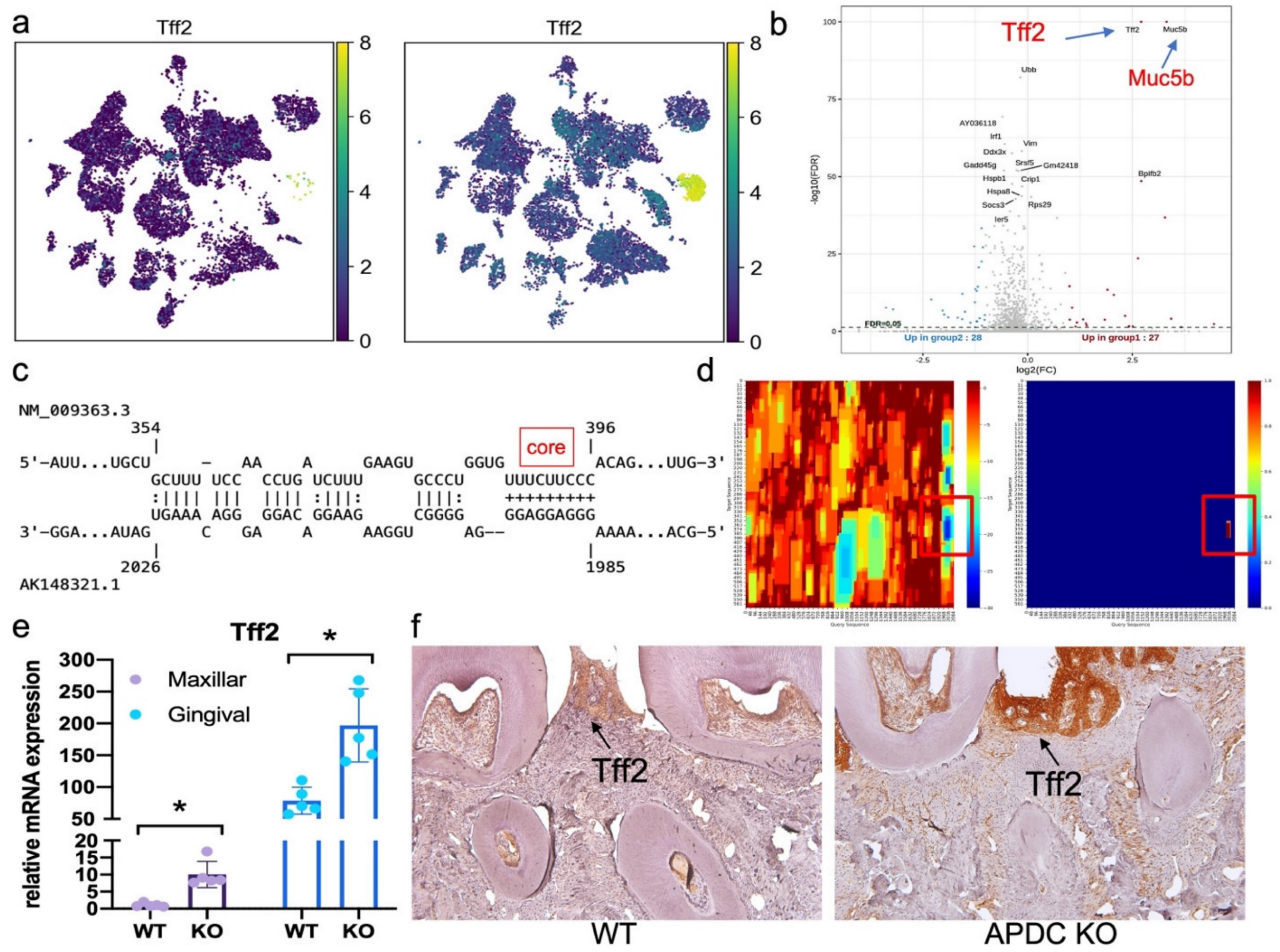
The mRNA and protein expression of Tff2 and its predicted binding site with APDC. a) the expression of Tff2 in WT and KO group across various cell types. b) the volcano plot illustrated the expression level of DEGs. c) the predicted binding site of Tff2 (top) and APDC (bottom). d) the heatmap showed the lowest binding energy and highest portability at the location of 1985 to 2026 on APDC. e) the Tff2 expression level in EP maxilla and gingival. f) the immunohistochemistry staining showed the Tff2 protein level in WT and KO groups.

Upon performing DEG gene ranking on all cell types in the gingival, we observed that the *Tff2* gene was the top upregulated gene across all cell types in the APDC-KO group Fig. S2 and S3). The UMP and volcano plot represent the differential expression levels of *Tff2* between the lncR-APDC-KO and wild-type groups (Fig. 6a and b). *Tff2* belongs to the trefoil factor family (TFFs) and is predominantly expressed in the stomach (50. Research regarding trefoil factors is an emerging area of investigation in the dental field. TFFs have been reported to be expressed in oral mucosal cells, with a novel role in chronic inflammatory conditions related to mucosal healing and function restoration. The TFFs expression have been detected in saliva and gingival samples presenting severe PD 51. To reveal the underlying mechanism between APDC deficiency and the high expression of *Tff2*, we further investigated the interaction of APDC and *Tff2* using IntaRNA; this tool enables accurate prediction of RNA-RNA hybrids by incorporating seed constraints and interaction site accessibility 52. The RNA sequence of APDC (AK148321, 2069 bp) and *Tff2* (NM_009363.3, 571 bp) were processed. The binding site with -25.07 kcal/mol interaction energy, close to 100% portability was found at the location of 1986 - 2026, close to 3' UTR of APDC (Fig. 6c and d), which indicates the direct interaction of the two RNAs. We proceeded to substantiate that the RNA expression level of *Tff2* was notably elevated in both maxillary bone and gingival tissues (Fig. 6e). Furthermore, we observed that TFF2 protein exhibited high expression levels specifically within the APDC-KO periodontitis samples (Fig. 6f).

### Cell-cell communications among different cell types in the inflamed gingival tissue

**Figure 7:**
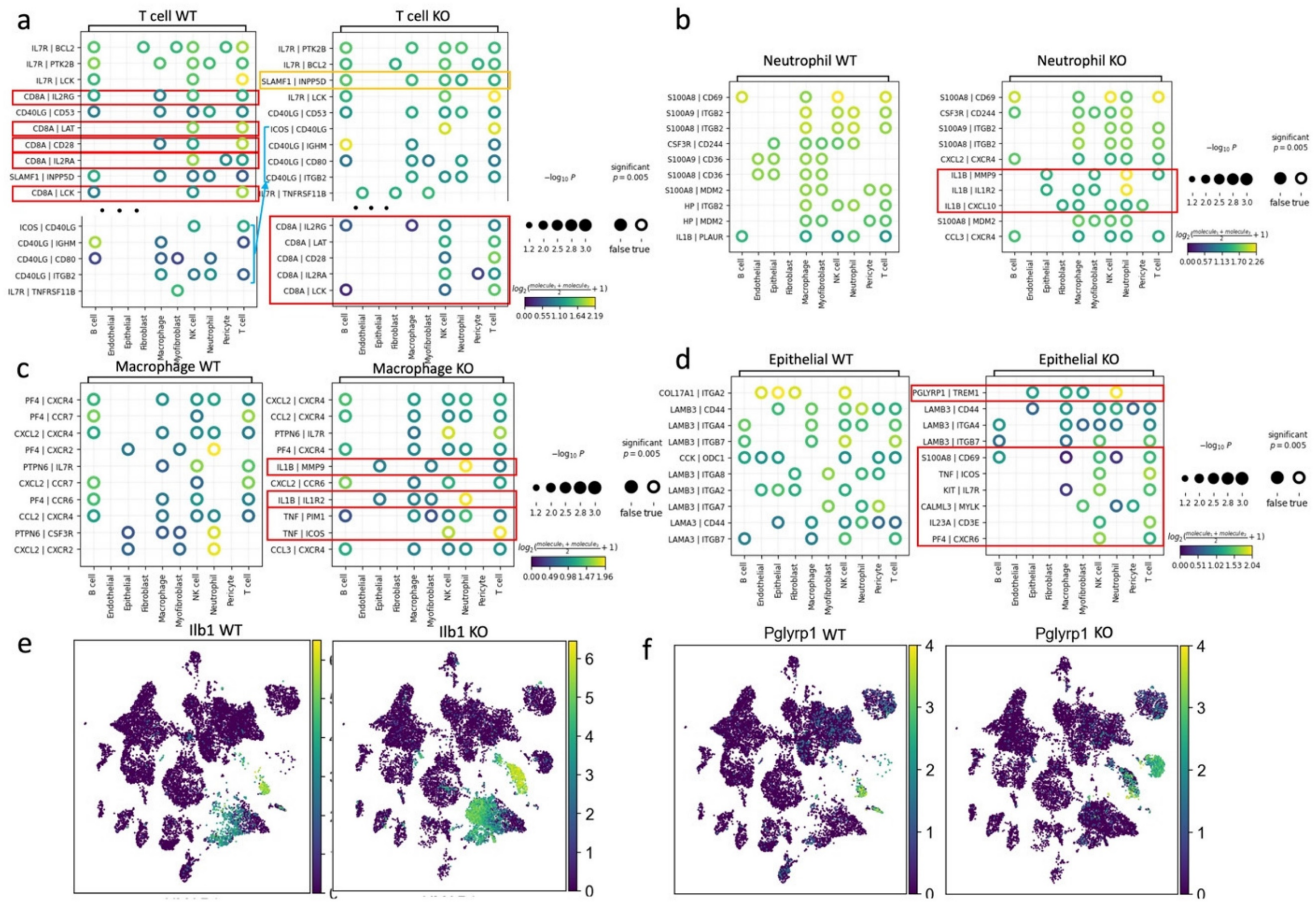
The cell-cell communications among different cell types. a) the plot results of top ligand-receptor pairs between T cell and other cell types in WT and KO group. b) the plot results of top ligand-receptor pairs between Neutrophil and other cell types in WT and KO group, c) the plot results of top ligand-receptor pairs between macrophage and other cell types in WT and KO group. d) the plot results of top ligand-receptor pairs between epithelial cells and other cell types in WT and KO group. e) The expression of Ilb1 in WT and KO respectively. f) The expression of Pglyrp1 in WT and KO respectively.

We performed a computational analysis to identify biologically relevant interacting ligand-receptor partners from our scRNA-seq data (see Methods). In the wild-type group, T cells communicated with B cells, macrophages, and NK cells through the ligand of CD8A. However, in the APDC-KO group, these interactions were significantly weakened, and the CD40 ligand (CD40LG) became the predominant mode of communication between T cells and other immune cells (Fig. 7a). These changes might be due to the lack of CD8+ cells in the APDC-KO group. In addition, the "SLAMF1-INPP5D" pair, associated with suppressed IFN-γ production in T-cells, showed a significant upregulation in the APDC-KO group.

Within the APDC-KO group, macrophages and neutrophils displayed heightened communication with T cells, epithelial cells, fibroblasts, and among themselves through IL1β (Fig. 7b and c). The KO macrophages and neutrophils consistently higher expressed IL1β compared to the wild type (Fig. 7e). In addition, the KO macrophages interacted with T and NK cells through TNF-PIM1 and TNF-ICOS (Fig. 5c). The PGLYRP1-TREM1 interaction was extremely active in the APDC-KO gingival tissue between epithelial cells and macrophages as well as neutrophils (Fig. 7d). Interestingly, Pglyrp1 was highly expressed in neutrophils and the subtype of epithelial cells (*Krt8+, Krt18+, Krt5-, Krt14-*) that we identified in the APDC-KO gingival (Fig. 7f). It has been shown that peptidoglycan recognition protein 1 (PGLYRP1) forms a Trem1 ligand complex with PGN, and stimulates cytokine production in neutrophils and macrophages 53. Furthermore, TREM1/PGLYRP1/IL1β signaling pathway regulation is related to the response to bacterial biofilm accumulation and removal in PD 54. Taken together, the upregulation of the TREM1/PGLYRP1/IL1β axis indicates a new mechanism describing how APDC deficiency causes a faster and more excessive immune response to the pathogen stimulation and leads to more severe periodontal inflammation and bone loss.

### AAV9-CAG-APDC significantly ameliorates periodontitis

**Figure 8:**
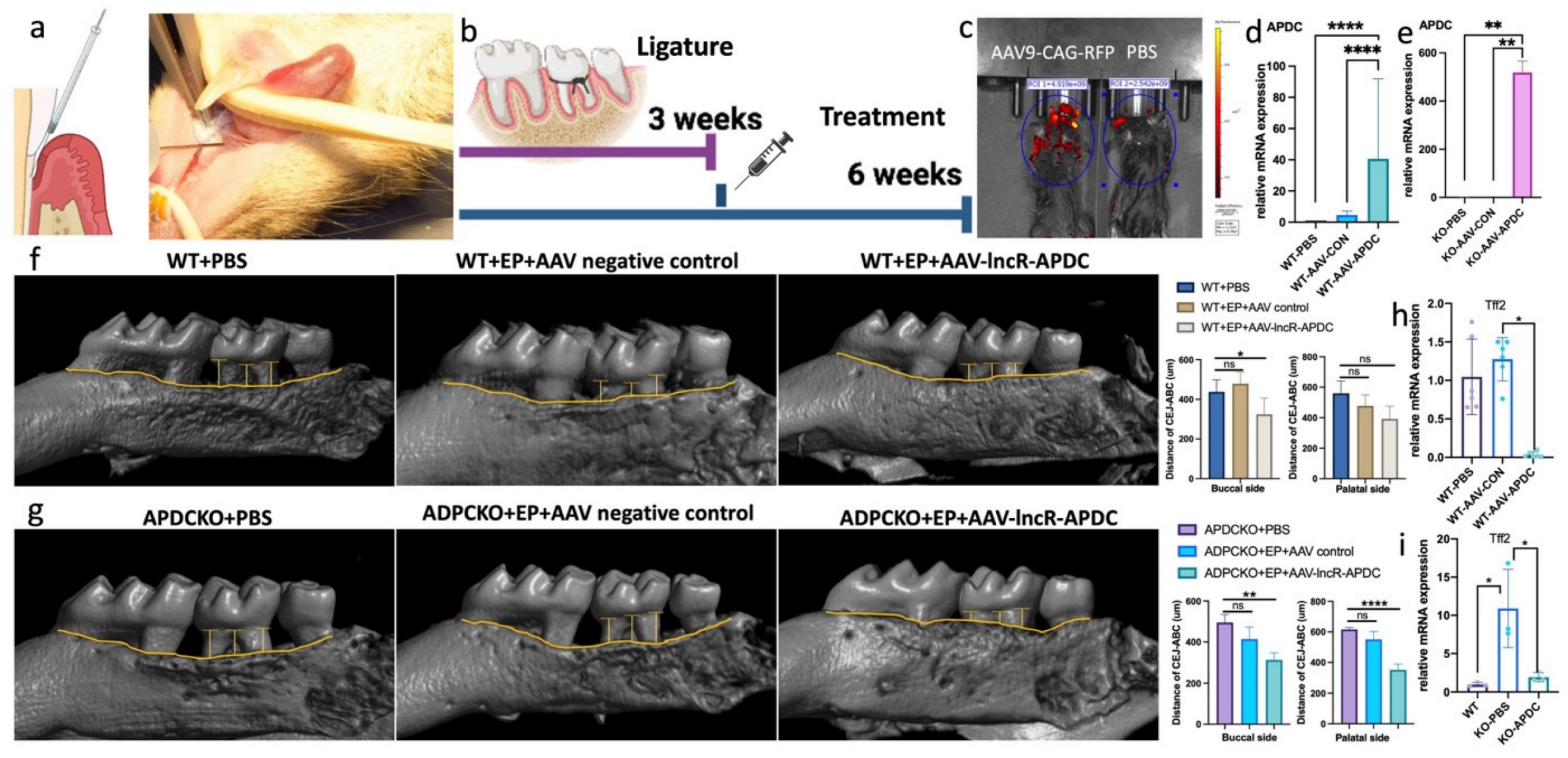
The AAV9-CAG-APDC attenuates periodontal bone destruction. a) the AAV-APDC was administrated through microinjection on gingiva. b) the timeline of the treatment. c) the IVIS imaging of the RFP expression in AAV and PBS group. d) the APDC expression level in wild type groups. e) the APDC expression level in APDC KO groups. f) Micro-CT showed the bone resorption level with and without AAV treatment in WT mice. g) Micro-CT showed the bone resorption level with and without AAV treatment in KO mice (n=4-6). h) The mRNA expression of Tff2 after AAV-APDC treatment in WT group. i) The mRNA expression of Tff2 after AAV-APDC treatment in KO group. * p < 0.05.

To assess the therapeutic impact of APDC in periodontitis, we constructed the adeno-associated virus 9-CAG-lncR-APDC (AAV9-APDC). EP was induced on 4-month-old female 129SVE (wild-type) and APDC-KO mice (6 groups, n=5-6 per group). The ligatures were removed after 3 weeks. AAV9-APDC was then administered through gingival microinjection to evaluate its ability to alleviate bone loss resulting from periodontitis. The AAV9 empty vector was used as the negative control (Fig. 8a and b). IVIS Spectrum CT imaging confirmed successful AAV9 delivery through local injection, as visualized by the expression of red fluorescent protein (RFP) (Fig. 8c). Furthermore, we evaluated the expression level of APDC and observed significantly elevated expression in the AAV9-APDC delivery groups (Fig. 8d and e). Remarkably, the WT-EP-AAV9-APDC group exhibited significant reduction in bone loss and a notable decrease in Tff2 level (Fig. 8f). Intriguingly, the alveolar bone loss in the KO-EP-AAV-APDC group was also significantly recovered and was close to the same level as the wild-type group with AAV-APDC treatment (Fig. 8g). Compared to the wild-type group, the *Tff2* level was considerably higher in the KO-PBS group. However, following the administration of AAV-APDC this elevation was notably reduced (Fig. 8h and i).

## Discussion

In the present study, we demonstrated that APDC deficiency exacerbates periodontitis by increasing alveolar bone loss and inflammatory infiltration of the periodontal tissue. The BMSCs harvested from lncR-APDC-KO mice showed downregulated osteogenic differentiation. Single cell RNA sequencing analysis of the gingival tissue from the EP model revealed that the deletion of lncR-APDC reduces the population of B cells and CD8+ cytotoxic T cells, while increasing the M1/M2 ratio and the recruitment of neutrophils to the inflamed periodontal tissues. Furthermore, a novel subtype of epithelial cells, characterized by a distinct gene profile (*Krt8*+ and *Krt18*+), and actively communicating with macrophages and neutrophils through the PGLYRP1-TREM1 pairing, was identified exclusively in the lncR-APDC-KO group. We also discovered that *Tff2* is the most highly expressed gene among the DEGs in all cell types of the inflamed lncR-APDC-KO gingival tissue, and predicted the direct binding of *Tff2* and lncR-APDC. Lastly, we successfully attenuated periodontal bone loss by delivering AAV9-CAG-APDC to the experimental periodontitis site, which shows the great potential of lncR-APDC in the treatment of periodontal disease.

After data filtering, we analyzed 25,161 single cells (13,244 from APDC-KO mice, and 11,917 from control mice), which is a satisfactory amount of high-quality data for scRNA seq. However, for the cell types with relatively small populations, there were still challenges when further dividing their subtypes. For example, we sub-clustered T cells into memory CD4 and CD8 T cells, as well as naïve CD4 and CD8 T cells based on the expression level of Cd4, Cd8a, Sell and Cd44. With more biological replicates, the enlarged pool of T cells would allow us to further divide the CD8+ T cells into Tc1, Tc2, Tc9, Tc17 and Tc22 T cells 55, and reveal more changes in the immune function of CD8+ T cells. Furthermore, NK cells are abundant in periodontitis lesions, and their activation has been causally linked to periodontal tissue destruction. In our scRNA seq analysis, NK cells were detected in both groups, though no significant difference was observed Fig. S2. In our future studies, we plan to acquire more data to thoroughly isolate and characterize these cell types. Additionally, we aim to integrate multi-omics approaches to comprehensively analyze the molecular signatures and signal pathways.

The wildly upregulated *Tff2* in the APDC deficient mice offers new insights into the functions of APDC. *Tff2* was originally found in the gastric mucosal lining, small and large intestine, oral mucosal cells, and salivary glands. At present, TFF expression has been detected in severe periodontal diseased tissue samples 51,56. Mahendra et al. reported markedly heightened levels of TFF2 protein in saliva samples from patients diagnosed with coronary heart disease (CHD) and PD 57. The discovery of the direct binding site of APDC and *Tff2* provides us a new direction to reveal APDC's regulatory mechanism in PD. In addition, the Mucin5b (*Muc5b*) gene was also significantly upregulated in many cell types in the APDC-KO group Fig. S2 and S3). This gene is specifically involved in mucosal innate immunity and is commonly co-expressed with the TFF family. We will perform further investigation into *Tff2*, *Muc5b* and related pathways in our forthcoming investigations.

Our study revealed that APDC deficiency leads to alterations in the interaction network between immune cells and other cell types in the gingival tissue. For example, we show that (1 the reduced interactions involving CD8A as a ligand (e.g., CD8A-IL2RA, CD8A-IL2RG and CD8A-CD28), between T cells and other immune cells, can be attributed to the absence of CD8+ cells in the APDC-KO group; and 2 the TREM1/PGLYRP1/IL1β axis is upregulated in the KO group and actively involved in epithelial cell, macrophage, and neutrophil interactions. Utilizing single-cell RNA sequencing data for ligand-receptor pairing analysis is a well-established method 58-60. Our results demonstrate the application of this method in studying periodontitis progression.

The lncRNAs have great potential in clinical application. There are currently 73 completed and ongoing clinical trials using lncRNAs as markers and therapeutic targets (clinicaltrials.gov). Notably, only two of these trials are focused on oral disease. Our understanding of the roles of lncRNAs in dental diseases is still in the early stages of exploration. The differentially expressed lncRNAs have been identified in oral lichen planus (OLP), oral submucous fibrosis (OSF), oral squamous cell carcinoma (OSCC), Sjӧgren's syndrome and periodontitis patients 61-67. However, the underlying epigenetic mechanisms governing the regulatory functions of these lncRNAs remain inconclusive. Furthermore, ANRIL is the shared risk factor of many chronic inflammatory diseases in humans. Besides CAD, PD, and diabetes, ANRIL is also involved in chronic obstructive pulmonary disease 68 and uric acid nephropathy 69. The analysis of APDC's regulatory role on immune response, intercellular communication among immune cells, and common pro-inflammation cytokines from this study, could provide fundamental knowledge for researching ANRIL and APDC related systemic inflammation diseases mentioned above.

This paper presents the first comprehensive study on the influence of lncR-APDC in bone resorption and immune response within inflamed periodontal tissue. It highlights changes in the ratio and functionality of immune and epithelial cells, and uncovers a distinctive epithelial subcluster and elevated *Tff2* expression related to APDC deficiency during periodontitis. The findings suggest that lncR-APDC holds significant potential for the prevention and treatment of PD, as well as other chronic inflammatory conditions.

In summary, this study suggests that incR-APDC is a critical player in the pathogenesis of PD and may offer therapeutic potential. Additionally, the identification of unique epithelial cell subsets and the interaction between lncR-APDC and *Tff2* open new avenues for understanding the epigenetic regulation of periodontitis. Further studies in this area could lead to innovative approaches for managing and treating PD.

## Methods

### The experimental periodontitis model

The 3D printing technology was applied in our modified experimental periodontitis surgery, which can dramatically reduce trauma and bleeding and shorten the duration of the procedure. The mice were anesthetized with ketamine (100mg/kg) and xylazine (10mg/kg) through intraperitoneal (IP) injection. Ligatures were held by the 3D-printed handle and locked tightly. We used a dental explorer to create a tiny gap between the molars and guide the suture. Ligatures were placed around the second molar and tied on the buccal side. After the EP surgery, the inserted ligatures were checked every 3 days and kept in place. All the animals that entered the study survived until the time point for sacrificing and sample collection. The periodontal tissue, alveolar bone and blood were collected. The 3D printed surgical tools were originally developed by Dr. Jiao's lab at the University of North Carolina at Chapel Hill 70. We modified the structure of the suture lock to extend its usage with the ability to apply ligature surrounding second molar. The tools were 3D printed with Tough 2000 Resin (Formlabs, Boston, MA) at the Hubs platform. All animals were housed at Tufts Comparative Medicine Services (CMS), with food and water provided ad libitum. A 12-hour light and 12-hour dark cycle was maintained during the experimental period.

### APDC knockout (KO) mouse model and genotyping

The APDC KO mouse model (129S6/SvEvTac-Del(4C4-C5)1Lap/Mmucd, MMRRC_032091-UCD) was obtained from the Mutant Mouse Resource & Research Center (MMRRC). In creating this model, lncRNA APDC (also known as Gm12610, AK148321) was deleted in W4/129S6 ES cells through Cre/loxP recombination. APDC was confirmed to be severely reduced in these chr4 Δ70kb/70kb mice 20. To further confirm the APDC-null genotypes of this strain, all APDC-KO and control mice enrolled in this study underwent confirmational genotyping. Tail DNA samples were extracted by DNeasy Blood & Tissue Kits, and genomic DNA genotyping analysis (QIAGEN, MD, USA) was performed with standard gDNA qPCR and gel electrophoresis. All animals with the same genotype same gender were randomized to the groups.

### Microcomputed tomography (μCT) analysis

The maxilla of the mice from the experimental and control group were scanned using an animal micro-computed tomography (μCT) system (SkyScan1172; Bruker‐microCT, Belgium) at Tufts Medical Center. The 3D models were then reconstructed from the raw images with Bruker NRecon and analyzed with software from Bruker, including CT Analyser 1.17.7.2, DataViewer 1.5.4.0 and CT Vox 3.3.0. The alveolar bone loss was described as the distance from the cementoenamel junction to the alveolar bone crest (CEJ‐ABC), and the values were measured at six periodontal sites (mesiobuccal, midbuccal, distobuccal, mesiopalatal, midpalatal, and distopalatal) of the second molars.

### Hemotoxylin and eosin (H&E) staining and TRAP staining of bone tissue

The left proximal tibia of APDC KO and control mice were fixed in 4% paraformaldehyde, embedded with paraffin, and cut into 5 μm sections. H&E staining and TRAP staining were performed according to the manufacturer's instructions. Digital images were taken with an OLYMPUS BX53 microscope (Olympus, Waltham, MA).

### Immunoassay

The serum samples were evaluated with the V-PLEX Plus Proinflammatory Panel1 Mouse Kit on the Meso Scale Discovery (MSD) MESO QuickPlex SQ 120 (MESO SCALE DIAGNOSTICS, Rockville, MD). The pro-inflammation cytokines: IFN-γ, IL-1β, IL-2, IL-4, IL-5, IL-6, KC/GRO, IL-10, IL-12p70, and TNF-α were quantitative determined.

### Cell culture

Bone marrow-derived mesenchymal cells (BMSCs) were harvested from six‐week‐old APDC KO and wild type mice. The mice were first killed by cervical dislocation following anesthesia respectively, and then sterilized with 70% (vol/vol) ethanol. The hind legs were removed from the body using scissors and all skin and muscle tissues were removed from the legs. The epiphyses of femur and tibia were cut off. The bone marrow was flushed out with Dulbecco's modified Eagle's medium (DMEM; Gibco® by Life Technologies^TM^) from the long bones, then cultured in the DMEM with 10% fetal bovine serum (FBS) and 1% penicillin/ streptomycin in a 5% CO_2_ incubator, at 37°C. After 24 hours, nonadherent cells were carefully removed and fresh medium was added. The BMSCs were treated with osteoblast differentiation medium (DM) including ascorbic acid (Asc, 50 μg/ml), dexamethasone (Dex, 0.1 μM) and β‐glycerophosphate (β‐Gly, 10 mM) for 3 days and differentiated to the osteoblasts (OBs).

### Next generation bulk RNA sequencing

The RNA library was prepared by Illumina Ribo-Zero Plus rRNA Depletion Kit followed by NEBNext® Ultra™ II RNA Library Prep Kit for Illumina®. The sequencing was performed on Illumina Novaseq 6000 S4 Flowcell (PE150). The minimum coverage per sample is 40M Read Pairs (12G). All samples passed the quality control (QC).

### RNA isolation and real-time polymerase chain reaction (RT-PCR)

The total RNA of tissues and cells was isolated using TRIzol (Thermo Fisher Scientific) and the Quick‐RNATM MiniPrep Kit (Zymo, CA). RNA of each sample was reverse‐transcribed by M‐MLV Reverse Transcriptase (Promega, WI) and cDNAs were quantified by RT-PCR with SYBR Green Supermix (Affymetrix) on Bio‐Rad iQ5 (Bio‐Rad Laboratories, CA).

### Preparation of single-cell suspensions

The gingival tissue surrounding the second molar was harvested after the ligature placement for 10 days from lncR-APDC knockout mice and control mice (n=4 per group). The gingival tissues were minced into small fragments (less than 1 mm^3^) by a #10 surgical blade. The minced sample was then transferred to a gentleMACS C tube (130-093-237, Miltenyi Biotech) with 10ml tissue culture media (RPMI with 2% FBS). C tubes were attached to the gentleMACS dissociator (130-093-235, Miltenyi Biotech) upside down, then performed program “m_lung_01.02”. Centrifuge C tube at 300 g for 5 mins. The supernatant was disposed and 10 mL of digestion solution (2 mg/ml type II collagenase, 2 mg/ml Dispase II, 0.04 mg/ml DNase in RPMI media) was added. The C tubes were incubated for 45 mins in a 37 ºC shaker. C tubes were attached to gentleMACS dissociator again and ran program “m_lung_02.01”. Samples were then centrifuged, resuspended with 0.5 ml tissue culture media, and filtered with 70 μm cell strainers. Fluorescence-activated Cell Sorting (FACS) was performed to remove the dead cells (3 μM DAPI) and increase the viability above 90%. After cell counting, samples with the final concentration of 700-1200 cells/μl were stored on ice until further processing. The whole procedure was performed on ice whenever possible.

### Single-cell RNA sequencing and data processing

Barcode specifically labeled the transcripts of each signal cell by using a Chromium Next GEM Single Cell 3' Kit v3.1 (10X Genomics Inc, San Francisco, USA). Libraries were constructed and sequenced on an Illumina NovaSeq 6000 platform (S10OD032203) at a sequencing depth of ~800 million reads in Tufts University Core Facility (TUCF). The sequencing results were demultiplexed and cell barcodes were extracted. Subsequently, Single-Cell Analysis in Python (SCANPY) 26 was utilized for data clustering analysis and visualization. SCSA 29, an automatic tool and distinct marker genes from published papers was used for cluster annotation. The ligand-receptor interaction data were generated by Squidpy 58 (based on CellPhoneDB 59 and Omnipath 60 database) via screening and paring the ligands and receptors, which expressed in more than 10% of cells in each cluster. The lncRNA-RNA reaction tool, intaRNA 52,71, was used to predict the direct bind site of lncR-APDC and Tff2. The newest intaRNA package was downloaded from GitHub to perform the analysis, which was able to analyze more than 2000 nt full length of lncRNAs and can be optimized on multiple settings.

### AAV delivery system

We designed the AAV9-CAG-APDC (Charles River Laboratories) for the delivery of APDC *in vivo*. The AAV9 with CAG promoter-driven expression of RFP (Charles River Laboratories, CV17176-AV9) was utilized to visualize the APDC delivery. The AAV9-CAG-APDC and control AAV vector were injected after 3 weeks of EP surgery. The animals were sacrificed after another 3 weeks.

### Statistics

All data are presented as means ± SD of results obtained from three or more experiments. The significance of differences in various categorical variables was evaluated using one‐way analysis of variance for multigroup comparisons and the t test for between two group comparisons. A p value of less than 0.05 was considered to be statistically significant. All statistical analyses were performed using SPSS statistics v27 (SPSS Inc., IL) and Prism GraphPad 9.0 (GraphPad Software).

## Supplementary Material

Supplementary figures.

## Abbreviations

EP: experimental periodontitis
BMSCs: bone marrow stem cells
CAD: coronary artery disease
PD: periodontal disease
ncRNA: Noncoding RNAs
lncRNAs: Long noncoding RNAs
hsCRP: high sensitive C-reactive protein
CEJ-ABC: distance from the cementoenamel junction to alveolar bone crest
DEG: differentially expressed genes
GO: gene ontology
PDLSCs: periodontal ligament stem cells
TFFs: trefoil factor family
Muc5b: Mucin5b
Pglyrp1: Peptidoglycan recognition protein 1
TREM1: Triggering Receptor Expressed On Myeloid Cells 1
RFP: Red Fluorescent Protein
OLP: oral lichen planus
OSF: oral submucous fibrosis
OSCC: oral squamous cell carcinoma
MMRRC: Mutant Mouse Resource & Research Center
μCT: micro-computed tomography
H&E: Hematoxylin and Eosin
IP: intraperitoneal
TRAP: tartrate resistant acid phosphatase
DMEM: Dulbecco's modified Eagle's medium
PCR: polymerase chain reaction
FACS: Fluorescence-activated Cell Sorting
scRNA-seq: Single Cell RNA Sequencing

## References

[1] Periodontal Disease | Oral Health Conditions | Division of Oral Health | CDC [Internet] 2018. Available from: https://www.cdc.gov/oralhealth/conditions/periodontal-disease.html.

[2] Abusleme L, Hoare A, Hong BY, Diaz PI (2021). Microbial signatures of health, gingivitis, and periodontitis. Periodontol 2000.

[3] Hajishengallis G, Chavakis T (2021). Local and systemic mechanisms linking periodontal disease and inflammatory comorbidities. Nat Rev Immunol.

[4] Sanz M, Marco Del Castillo A, Jepsen S, Gonzalez-Juanatey JR, D'Aiuto F, Bouchard P (2020). Periodontitis and cardiovascular diseases: Consensus report. J Clin Periodontol.

[5] Liccardo D, Cannavo A, Spagnuolo G, Ferrara N, Cittadini A, Rengo C (2019). Periodontal Disease: A Risk Factor for Diabetes and Cardiovascular Disease. Int J Mol Sci.

[6] Kinane DF, Stathopoulou PG, Papapanou PN (2017). Periodontal diseases. Nat Rev Dis Primers.

[7] Peng WX, Koirala P, Mo YY (2017). LncRNA-mediated regulation of cell signaling in cancer. Oncogene.

[8] Huang Y (2018). The novel regulatory role of lncRNA-miRNA-mRNA axis in cardiovascular diseases. J Cell Mol Med.

[9] Rahimi E, Ahmadi A, Boroumand MA, Mohammad Soltani B, Behmanesh M (2018). Association of ANRIL Expression with Coronary Artery Disease in Type 2 Diabetic Patients. Cell J.

[10] Kong Y, Hsieh CH, Alonso LC (2018). ANRIL: A lncRNA at the CDKN2A/B Locus With Roles in Cancer and Metabolic Disease. Frontiers in Endocrinology [Internet]. 2018;9. Available from: https://www.frontiersin.org/articles/10.3389/fendo.

[11] Razeghian-Jahromi I, Karimi Akhormeh A, Zibaeenezhad MJ (2022). The Role of ANRIL in Atherosclerosis. Disease Markers.

[12] Bochenek G, Häsler R, El Mokhtari NE, König IR, Loos BG, Jepsen S (2013). The large non-coding RNA ANRIL, which is associated with atherosclerosis, periodontitis and several forms of cancer, regulates ADIPOR1, VAMP3 and C11ORF10. Hum Mol Genet.

[13] Schaefer AS, Richter GM, Groessner-Schreiber B, Noack B, Nothnagel M, El Mokhtari NE (2009). Identification of a shared genetic susceptibility locus for coronary heart disease and periodontitis. PLoS Genet.

[15] Santos CMML, Lira-Junior R, Fischer RG, Santos APP, Oliveira BH (2015). Systemic Antibiotics in Periodontal Treatment of Diabetic Patients: A Systematic Review. PLoS One.

[16] Gholami L, Ghafouri-Fard S, Mirzajani S, Arsang-Jang S, Taheri M, Dehbani Z (2020). The lncRNA ANRIL is down-regulated in peripheral blood of patients with periodontitis. Noncoding RNA Res.

[17] Schaefer AS, Richter GM, Dommisch H, Reinartz M, Nothnagel M, Noack B (2011). CDKN2BAS is associated with periodontitis in different European populations and is activated by bacterial infection. Journal of Medical Genetics.

[18] Liu X, Zhou Y (2021). Downregulation of lncRNA ANRIL Inhibits Osteogenic Differentiation of Periodontal Ligament Cells via Sponging miR-7 through NF-κB Pathway. Anal Cell Pathol (Amst).

[19] Teeuw WJ, Laine ML, Bizzarro S, Loos BG (2015). A Lead ANRIL Polymorphism Is Associated with Elevated CRP Levels in Periodontitis: A Pilot Case-Control Study. PLOS ONE.

[20] Visel A, Zhu Y, May D, Afzal V, Gong E, Attanasio C (2010). Targeted deletion of the 9p21 non-coding coronary artery disease risk interval in mice. Nature.

[21] Gao S, Cheng QC, Hu YG, Tan ZZ, Chen L, Liu SW (2021). LncRNA AK148321 alleviates neuroinflammation in LPS-stimulated BV2 microglial cell through regulating microRNA-1199-5p/HSPA5 axis. Life Sciences.

[22] Loinard C, Basatemur G, Masters L, Baker L, Harrison J, Figg N (2014). Deletion of Chromosome 9p21 Noncoding Cardiovascular Risk Interval in Mice Alters Smad2 Signaling and Promotes Vascular Aneurysm. Circulation: Cardiovascular Genetics.

[23] Pan W, Wang Q, Chen Q (2019). The cytokine network involved in the host immune response to periodontitis. Int J Oral Sci.

[24] Ramadan DE, Hariyani N, Indrawati R, Ridwan RD, Diyatri I (2020). Cytokines and Chemokines in Periodontitis. Eur J Dent.

[25] Behl Y, Siquiera M, Ortiz J, Desta T, Faibish D, Graves DT (2008). Activation of the Acquired Immune Response Reduces Coupled Bone Formation in Response to a Periodontal Pathogen. J Immunol.

[26] Wolf FA, Angerer P, Theis FJ (2018). SCANPY: large-scale single-cell gene expression data analysis. Genome Biology.

[27] Chen Y, Wang H, Yang Q, Zhao W, Chen Y, Ni Q (2022). Single-cell RNA landscape of the osteoimmunology microenvironment in periodontitis. Theranostics.

[28] Williams DW, Greenwell-Wild T, Brenchley L, Dutzan N, Overmiller A, Sawaya AP (2021). Human oral mucosa cell atlas reveals a stromal-neutrophil axis regulating tissue immunity. Cell.

[29] Cao Y, Wang X, Peng G (2020). SCSA: A Cell Type Annotation Tool for Single-Cell RNA-seq Data. Frontiers in Genetics [Internet]. 2020;11. Available from: https://www.frontiersin.org/article/10.3389/fgene.

[30] Alvarez C, Abdalla H, Suliman S, Rojas P, Wu YC, Almarhoumi R (2021). RvE1 Impacts the Gingival Inflammatory Infiltrate by Inhibiting the T Cell Response in Experimental Periodontitis. Frontiers in Immunology [Internet]. 2021;12. Available from: https://www.frontiersin.org/articles/10.3389/fimmu.

[31] Golubovskaya V, Wu L (2016). Different Subsets of T Cells, Memory, Effector Functions, and CAR-T Immunotherapy. Cancers (Basel).

[32] Sckisel GD, Mirsoian A, Minnar CM, Crittenden M, Curti B, Chen JQ (2017). Differential phenotypes of memory CD4 and CD8 T cells in the spleen and peripheral tissues following immunostimulatory therapy. Journal for ImmunoTherapy of Cancer.

[33] Cardoso EM, Arosa FA (2017). CD8+ T Cells in Chronic Periodontitis: Roles and Rules. Front Immunol.

[34] Choi Y, Woo KM, Ko SH, Lee YJ, Park SJ, Kim HM (2001). Osteoclastogenesis is enhanced by activated B cells but suppressed by activated CD8(+) T cells. Eur J Immunol.

[35] Cyster JG, Allen CDC (2019). B cell responses - Cell interaction dynamics and decisions. Cell.

[36] Figueredo CM, Lira-Junior R, Love RM (2019). T and B Cells in Periodontal Disease: New Functions in A Complex Scenario. Int J Mol Sci.

[37] Shen Y, Ma Y, Xie J, Lin L, Shi Y, Li X (2020). A regulatory role for CD72 expression on B cells and increased soluble CD72 in primary Sjogren's syndrome. BMC Immunology.

[38] Spivia W, Magno PS, Le P, Fraser DA (2014). Complement protein C1q promotes macrophage anti-inflammatory M2-like polarization during the clearance of atherogenic lipoproteins. Inflamm Res.

[39] Han Y, Huang Y, Gao P, Yang Q, Jia L, Zheng Y (2022). Leptin Aggravates Periodontitis by Promoting M1 Polarization via NLRP3. J Dent Res.

[40] Rosales C (2018). Neutrophil: A Cell with Many Roles in Inflammation or Several Cell Types? Frontiers in Physiology [Internet]. 2018;9. Available from: https://www.frontiersin.org/articles/10.3389/fphys.

[41] Jiang Q, Zhao Y, Shui Y, Zhou X, Cheng L, Ren B (2021). Interactions Between Neutrophils and Periodontal Pathogens in Late-Onset Periodontitis. Frontiers in Cellular and Infection Microbiology [Internet]. 2021;11. Available from: https://www.frontiersin.org/articles/10.3389/fcimb.

[42] Mortaz E, Alipoor SD, Adcock IM, Mumby S, Koenderman L (2018). Update on Neutrophil Function in Severe Inflammation. Front Immunol.

[43] Del Prete A, Martínez-Muñoz L, Mazzon C, Toffali L, Sozio F, Za L (2017). The atypical receptor CCRL2 is required for CXCR2-dependent neutrophil recruitment and tissue damage. Blood.

[44] Qian S jiao, Huang Q ru, Chen R ying, Mo J ji, Zhou L yi, Zhao Y (2021). Single-Cell RNA Sequencing Identifies New Inflammation-Promoting Cell Subsets in Asian Patients With Chronic Periodontitis. Front Immunol.

[45] Smith PC, Martínez C, Martínez J, McCulloch CA (2019). Role of Fibroblast Populations in Periodontal Wound Healing and Tissue Remodeling. Front Physiol.

[46] Smith P (2018). Role of myofibroblasts in normal and pathological periodontal wound healing. Oral Diseases.

[47] Darby IA, Laverdet B, Bonté F, Desmoulière A (2014). Fibroblasts and myofibroblasts in wound healing. Clin Cosmet Investig Dermatol.

[48] Komaki M (2019). Pericytes in the Periodontal Ligament. Adv Exp Med Biol.

[49] Han W, Hu C, Fan ZJ, Shen GL (2021). Transcript levels of keratin 1/5/6/14/15/16/17 as potential prognostic indicators in melanoma patients. Sci Rep.

[50] Hoffmann W (2020). Trefoil Factor Family (TFF) Peptides and Their Diverse Molecular Functions in Mucus Barrier Protection and More: Changing the Paradigm. Int J Mol Sci.

[51] Choudhary A, Smitha CN, Suresh DK (2015). Trefoils: An unexplored natural protective shield of oral cavity. J Oral Biol Craniofac Res.

[52] Mann M, Wright PR, Backofen R (2017). IntaRNA 2.0: enhanced and customizable prediction of RNA-RNA interactions. Nucleic Acids Research.

[53] Read CB, Kuijper JL, Hjorth SA, Heipel MD, Tang X, Fleetwood AJ (2015). Cutting Edge: Identification of Neutrophil PGLYRP1 as a Ligand for TREM-1. The Journal of Immunology.

[54] Silbereisen A, Hallak AK, Nascimento GG, Sorsa T, Belibasakis GN, Lopez R (2019). Regulation of PGLYRP1 and TREM-1 during Progression and Resolution of Gingival Inflammation. JDR Clinical & Translational Research.

[55] Paul MS, Ohashi PS (2020). The Roles of CD8+ T Cell Subsets in Antitumor Immunity. Trends in Cell Biology.

[56] Chaiyarit P, Chayasadom A, Wara-Aswapati N, Hormdee D, Sittisomwong S, Nakaresisoon S (2012). Trefoil factors in saliva and gingival tissues of patients with chronic periodontitis. J Periodontol.

[57] Mahendra J, Srinivasan S, Kanakamedala A, D N, Namasivayam A, Mahendra L (2023). Expression of trefoil factor 2 and 3 and adrenomedullin in chronic periodontitis subjects with coronary heart disease. J Periodontol.

[58] Palla G, Spitzer H, Klein M, Fischer D, Schaar AC, Kuemmerle LB (2022). Squidpy: a scalable framework for spatial omics analysis. Nat Methods.

[59] Efremova M, Vento-Tormo M, Teichmann SA, Vento-Tormo R (2020). CellPhoneDB: inferring cell-cell communication from combined expression of multi-subunit ligand-receptor complexes. Nat Protoc.

[60] Türei D, Korcsmáros T, Saez-Rodriguez J (2016). OmniPath: guidelines and gateway for literature-curated signaling pathway resources. Nat Methods.

[61] Wang YK, Liu CM, Lin T, Fang CY, Yu CC, Yu CH (2020). Inhibition of HIF1A-AS1 impedes the arecoline-induced migration activity of human oral mucosal fibroblasts. J Formos Med Assoc.

[62] Yang Q, Xu B, Sun H, Wang X, Zhang J, Yu X (2017). A genome-wide association scan of biological processes involved in oral lichen planus and oral squamous cell carcinoma. Medicine (Baltimore).

[63] Wang J, Zhai X, Guo J, Li Y, Yang Y, Wang L (2019). Long non-coding RNA DQ786243 modulates the induction and function of CD4+ Treg cells through Foxp3-miR-146a-NF-κB axis: Implications for alleviating oral lichen planus. Int Immunopharmacol.

[64] Zhang L, Meng X, Zhu XW, Yang DC, Chen R, Jiang Y (2019). Long non-coding RNAs in Oral squamous cell carcinoma: biologic function, mechanisms and clinical implications. Mol Cancer.

[65] Zou Y, Li C, Shu F, Tian Z, Xu W, Xu H (2015). lncRNA expression signatures in periodontitis revealed by microarray: the potential role of lncRNAs in periodontitis pathogenesis. J Cell Biochem.

[66] Dolcino M, Tinazzi E, Vitali C, Del Papa N, Puccetti A, Lunardi C (2019). Long Non-Coding RNAs Modulate Sjögren's Syndrome Associated Gene Expression and Are Involved in the Pathogenesis of the Disease. J Clin Med.

[67] Zhang K, Qiu W, Wu B, Fang F (2020). Long non-coding RNAs are novel players in oral inflammatory disorders, potentially premalignant oral epithelial lesions and oral squamous cell carcinoma (Review). Int J Mol Med.

[68] Ge J, Geng S, Jiang H (2019). Long noncoding RNAs antisense noncoding RNA in the INK4 locus (ANRIL) correlates with lower acute exacerbation risk, decreased inflammatory cytokines, and mild GOLD stage in patients with chronic obstructive pulmonary disease. J Clin Lab Anal.

[69] Hu J, Wang D, Wu H, Yang Z, Yang N, Dong J (2019). Long non-coding RNA ANRIL-mediated inflammation response is involved in protective effect of rhein in uric acid nephropathy rats. Cell Biosci.

[70] Marchesan J, Girnary MS, Jing L, Miao MZ, Zhang S, Sun L (2018). An experimental murine model to study periodontitis. Nat Protoc.

[71] Busch A, Richter AS, Backofen R (2008). IntaRNA: efficient prediction of bacterial sRNA targets incorporating target site accessibility and seed regions. Bioinformatics.

