# Supplementary material for: A novel lncRNA-mediated epigenetic regulatory mechanism in periodontitis
Source: Int J Biol Sci. 2023 Oct 9;19(16):5187–203. doi: 10.7150/ijbs.87977 (PMC10620817; doi:10.7150/ijbs.87977)
Supplement: Supplementary file 1 — Supplementary figures. [file ijbsv19p5187s1.pdf]

Supplementary materials

Supplementary Fig. 1

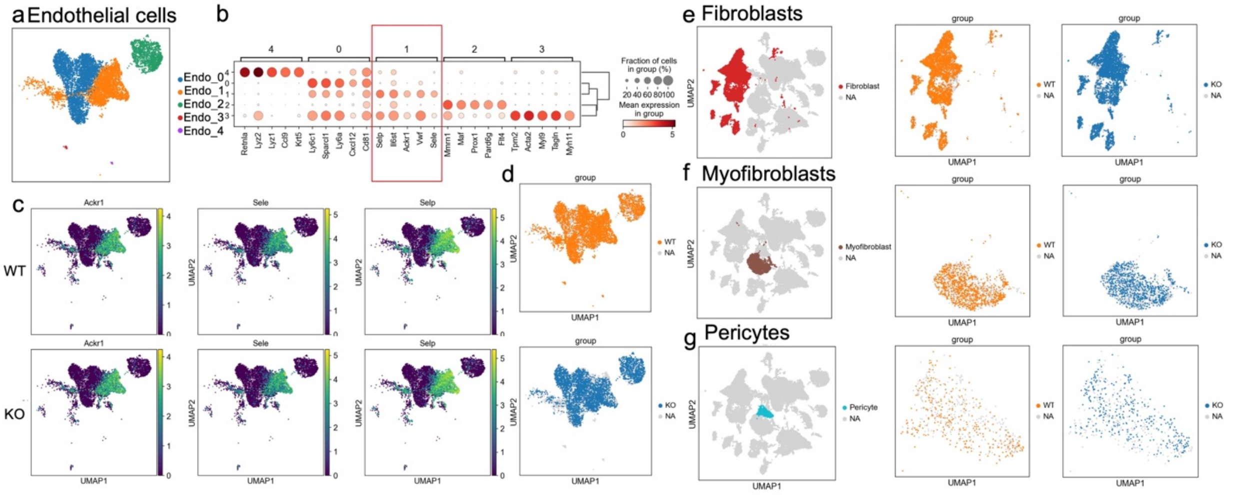

S Fig. 1 Endothelial cells, fibroblasts, myofibroblasts and pericytes are in the inflamed tissues of both groups. (a)Endothelial cells are the biggest cellular component in the inflamed gingival samples. Total of 5 subclusters were identified. (b)Subcluster_1, which highly expressed genes, Selp, Ackr1, and Sele, are reported to be highly associated with immune regulation during PD development and indicates the tissue samples are inflamed. (c,d)We further checked the related gene expression and cell counts in both KO and WT group. There is no significant disparity between two groups. (e-g)Fibroblasts are centrally involved in the wound healing response and remodeling of the periodontal tissue. During the remodeling phase of wound healing, a specific subtype of fibroblast may emerge, which is the Myofibroblast. Myofibroblasts may also derive from alternative sources, including mesenchymal stem cells, pericytes, and epithelial cells. It was recently reported that pericytes possess multilineage differentiation capacity and can be the source of tissue stem cells and/or progenitor cells, making them similar to periodontal ligament stem cells (PDLSCs). In our data, the gene profiling and cell counts for these three cell populations are consistent with other periodontitis related studies and did not show a disparity between APDC KO and wildtype group.

Supplementary Fig. 2

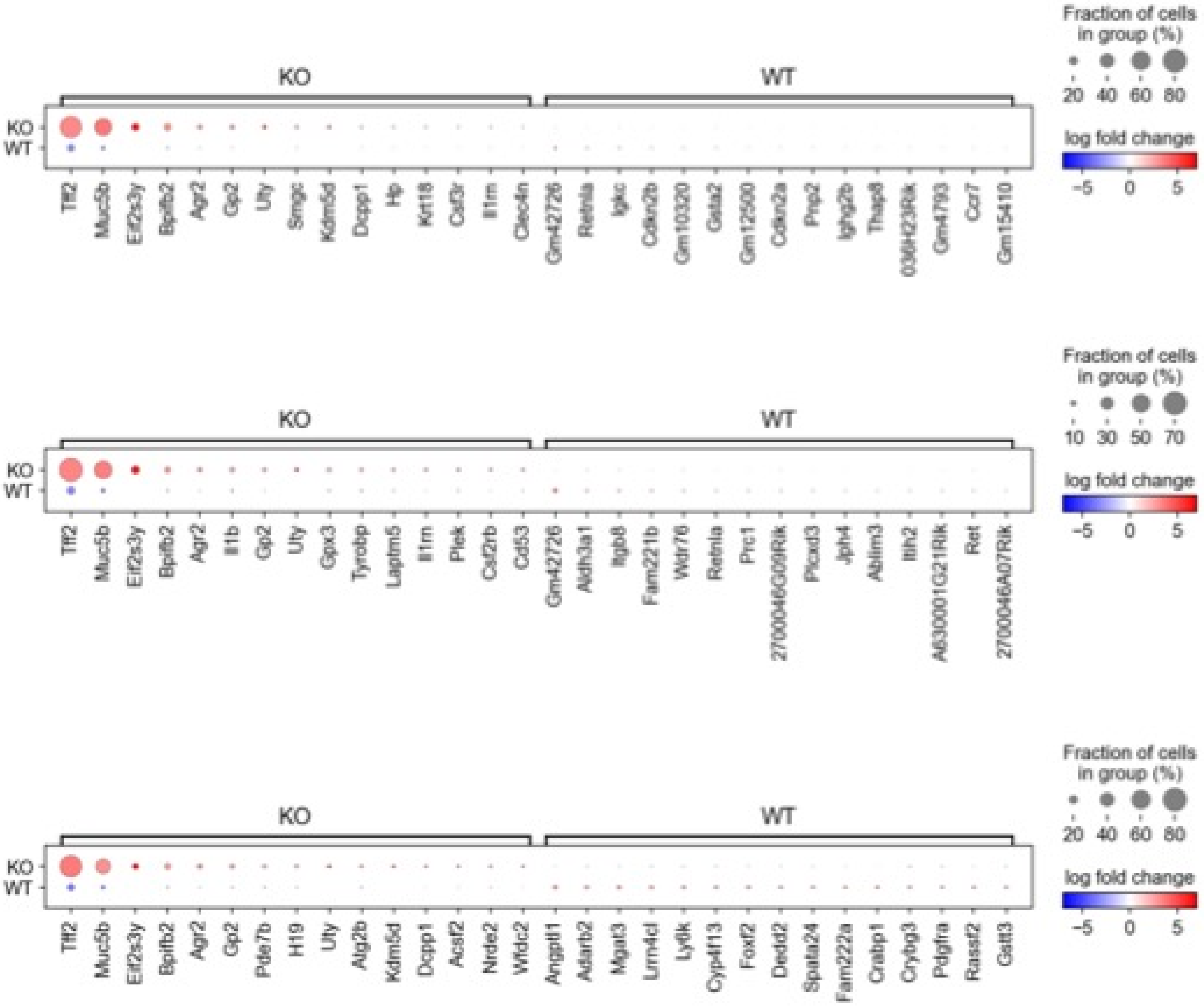

S Fig. 2 The DEGs analysis of the B cells, endothelial cells, epithelial cells, macrophages, NK cells, Neutrophils and T cells.

Supplementary Fig. 3.

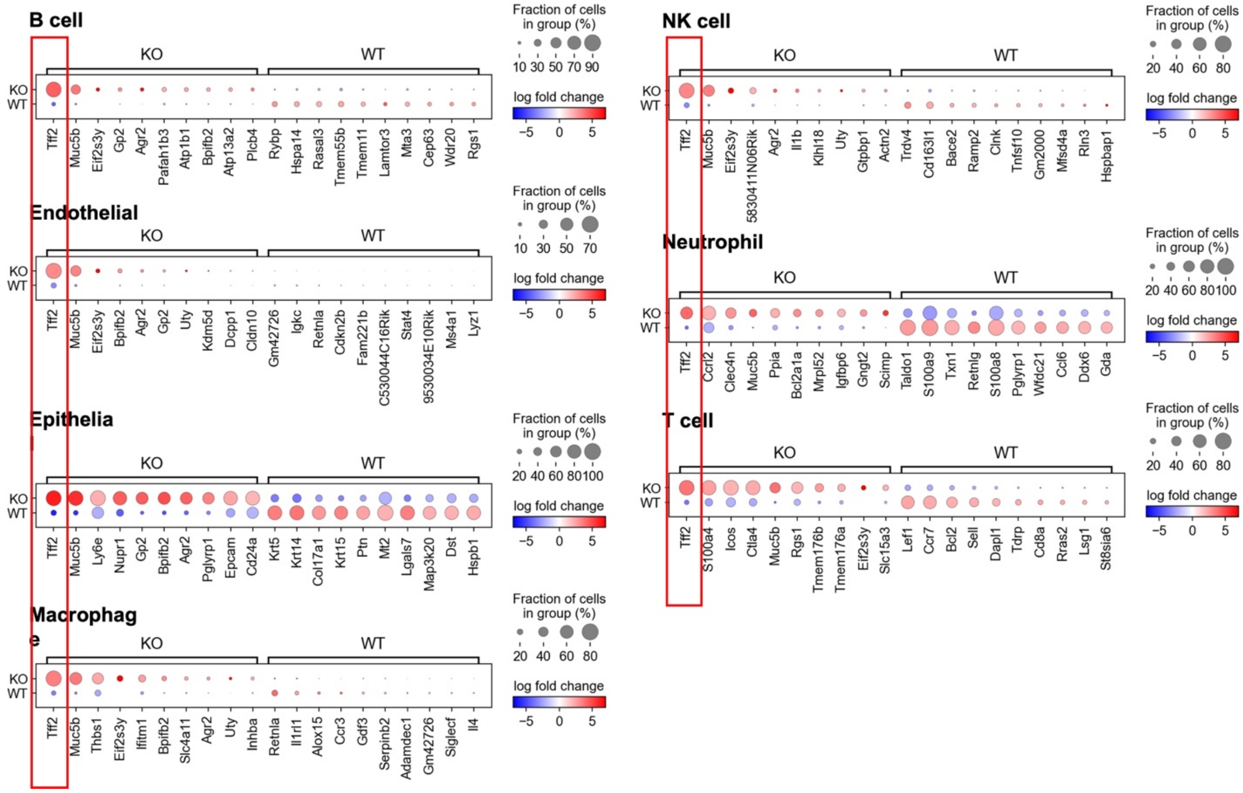

S Fig. 3 The DEGs analysis of the fibroblasts, myofibroblasts and pericytes.
